# A general consonance principle for closure tests based on p -values

**DOI:** 10.1177/09622802241269624

**Published:** 2024-10-23

**Authors:** Sonja Zehetmayer, Franz Koenig, Martin Posch

**Affiliations:** 1 Center for Medical Data Science, Medical University of Vienna, Austria

**Keywords:** Combination test, closed test, multiple testing, Stouffer test, Fisher combination test

## Abstract

The closure principle is a powerful approach to constructing efficient testing procedures controlling the familywise error rate in the strong sense. For small numbers of hypotheses and the setting of independent elementary p -values we consider closed tests where each intersection hypothesis is tested with a p -value combination test. Examples of such combination tests are the Fisher combination test, the Stouffer test, the Omnibus test, the truncated test, or the Wilson test. Some of these tests, such as the Fisher combination, the Stouffer, or the Omnibus test, are not consonant and rejection of the global null hypothesis does not always lead to rejection of at least one elementary null hypothesis. We develop a general principle to uniformly improve closed tests based on p -value combination tests by modifying the rejection regions such that the new procedure becomes consonant. For the Fisher combination test and the Stouffer test, we show by simulations that this improvement can lead to a substantial increase in power.

## 1. Introduction

When several elementary null hypotheses are tested simultaneously, an adjustment for multiple testing is required to control an overall error rate. For example, in a clinical trial often several endpoints, treatment groups, or subgroups are considered and control of the familywise error rate (FWER) in the strong sense is desired, which means that the probability of rejecting at least one true null hypothesis must not exceed the pre-specified significance level α under any configuration of true and false null hypotheses.

To control the FWER in the strong sense, different approaches have been suggested^1^: one group of methods is based on minimum *p*-value statistics. The *p*-values may be ordered and individual significance boundaries are derived, as, for example, for the Bonferroni or the Hommel^2^ procedure. A second group of methods is based on combinations of p -values, for example, the Fisher combination test,^3^ truncated test,^4^ Stouffer z -score,^5^ or Omnibus test.^6^ Often, such combination tests are considered as tests for the global null hypothesis, however, with the closure testing principle also elementary hypothesis tests can be performed: the closure testing principle^7^ is a general concept to construct multiple testing procedures with strong FWER control and it rejects an elementary null hypothesis if all intersection hypotheses (including the global null hypothesis) of subsets containing it are rejected using some combination test. A prominent example is the closure principle applied to the Bonferroni test, which leads to the Bonferroni–Holm procedure.^8^ Note that when testing multiple hypotheses, in general no uniformly most powerful test exists,^1^ as the performance of the test depends not only on the power definition^9^ used and number of hypotheses tested, but also on unknown parameters as effect sizes or ratio of true alternative hypotheses.

Some of the considered combination tests, as, for example, the Stouffer test,^5^ the Fisher combination test,^3^ or the Omnibus test (for log-transformed p -values)^6^ are not consonant with the closure testing principle, which means that rejection of the global null hypothesis does not always lead to rejection of at least one elementary null hypothesis. Lack of consonance, however, not only can hamper the interpretation of the results, but also leads to strictly conservative tests for the elementary hypotheses. Therefore modifications of non-consonant procedures have been proposed^10–12^ to guarantee consonance by removing parts of the rejection regions when testing the intersection hypotheses, that do not result in rejection of any of the contained elementary null hypotheses. To compensate for this reduction, the rejection region is shifted to other regions (such that the multiple testing procedure remains consonant) to exhaust the level α . For example, for multivariate normal test statistics with a unit-variance compound symmetric covariance matrix, Bittman et al.^13^ constructed optimal consonant tests and showed that maximin optimal tests for intersection null hypotheses can be uniformly improved for different correlations. Romano et al.^11^ extended this approach for two-sided tests and showed that for all non-consonant tests, there exists “a consonant test that reaches the same decision for the hypotheses of interest as the original procedure” (Theorem 3.1 in Romano et al.^11^). However, the consonant test might be strictly conservative. Romano et al.^11^ showed maximin optimality of the closed test, if the tests for all (intersection) hypotheses are maximin optimal and the closed test is consonant (Theorem 4.1 in Romano et al.^11^). For the case of two elementary null hypotheses, they further showed a result (Theorem A1 in Romano et al.^11^) that allows to construct a maximin optimal test also in the case where the maximin optimal test for the intersection hypothesis is not consonant.

Niewczas et al.^10^ considered optimal testing of one-sided hypotheses that are bivariate normally distributed with known correlation and extended the method of Bittman et al.^13^ for unequal treatment effects. Further work on consonant and optimal tests can be found in the literature.^14–19^

In this article, we propose a general principle to construct consonant closed tests of intersection hypothesis tests based on symmetric p -value combination tests. We show that the proposed tests partially achieve a large increase in average power – defined as the proportion of correctly rejected alternatives among all alternatives – compared to closed tests that directly test intersection hypotheses using the combination tests. This improvement becomes larger with an increasing number of null hypotheses, in particular, for the Stouffer test.

In the next section, we review the closure principle and combination tests. Then we introduce an approach to construct consonant closed tests with intersection hypothesis tests based on combination tests and discuss a shortcut. In the Results section, we report the results of a simulation study comparing the Fisher combination test, Omnibus test, Stouffer test, harmonic mean test,^20^ truncated test,^4^ Hommel procedure, Gou procedure^21^ and Bonferroni–Holm test for a large number of scenarios. Special focus lies on the comparison with the corresponding consonant closed test based on the Fisher combination test, Stouffer test, Omnibus test with log-transformed p -values, and Hommel test. The methods are illustrated with two real data examples from clinical trials where treatment effects in disjoint subgroups were tested.

R-programs implementing the consonant closed test for the Stouffer test and the Fisher combination test are available for a maximum number of 10 hypotheses and for the Omnibus test with log transformation for a maximum number of six hypotheses (see https://github.com/SonjaZehetmayer/consonance). This repository contains R functions to reproduce the results of the simulation study and the simulated null distributions.

## 2. Multiple testing procedures based on combinations of p -values

Consider m elementary null hypotheses $H_{1},\ldots ,H_{m}$ and assume that for each null hypothesis $H_{i}$ a test statistic $T_{i}$ and a corresponding elementary p -value $p_{i}$ , i∈{1,…,m} , are defined.

### 2.1. The closure test

The closure principle^22^ is a general method which allows to make simultaneous conclusions about elementary null hypotheses $H_{i},i\in \text{M}=\{1,\ldots ,m\}$ while controlling strongly the FWER at a pre-specified level α . The closure principle requires that for all possible intersection hypotheses $H_{L}=\underset{i\in L}{⋂}H_{i}$ , L⊆M , a hypothesis test is defined. Note that this includes also the elementary hypotheses $H_{i}$ (where |L|=1 and |L| denotes the cardinality of the set L ) and the global null hypothesis, $H_{\text{M}}=\overset{m}{\underset{i=1}{⋂}}H_{i}$ . All elementary and intersection hypotheses are tested using such a local level α test. An elementary hypothesis $H_{i}$ can be rejected by controlling the FWER at level α , if all intersection hypotheses of sets L that include i can be rejected by their local test at level α . For example, in the case of two elementary null hypotheses $H_{1}$ and $H_{2}$ , $H_{1}$ can only be rejected if $H_{\left\{1,2\right\}}=H_{1}\cap H_{2}$ as well as $H_{1}$ can be rejected with the local tests. If $H_{\left\{1,2\right\}}$ is not rejected by its local test, no elementary hypothesis can be rejected according to the closure principle. Any test which controls the local level may be used to test the intersection hypotheses, making this method very general.

## 3. Consonant tests

A closed testing method is called consonant, if the rejection of an intersection hypothesis implies the rejection of at least one of its elementary null hypotheses in the closed test,^22,23^ for example, for m=2 , rejection of $H_{\left\{1,2\right\}}$ must imply rejection of $H_{1}$ , $H_{2},$ or both of them. Formally, a closed testing procedure is consonant^11^ if the rejection of an intersection hypothesis $H_{L}$ with L⊆M and |L|>1 always leads to the rejection of at least one $H_{J}$ implied by $H_{L}$ , that is, $H_{J}$ with J⊂L .

Below we give three examples of intersection hypothesis tests which are not consonant when applied in a closed test.

*Fisher combination test*. This test^3^ for the global null hypothesis combines the one-sided log-transformed p -values $p_{1},\ldots ,p_{m}$ by taking the sum to obtain the test statistic $T^{F}=-\sum_{i=1}^{m}2\log\;p_{i}$ . Under the assumption of independent uniformly distributed *p*-values, the null distribution is $T^{F}∼\chi_{2m}^{2}$ .

*Stouffer test (Stouffer’s z -test)*. Based on $Z_{i}=z_{1-p_{i}}$ , where $z_{1-p_{i}}$ denotes the $1-p_{i}$ quantile of the standard normal distribution, the combined test statistic of the Stouffer test^5^ (also called inverse normal test) is given by $T^{S}=\sum_{i=1}^{m}Z_{i}/\sqrt{m}$ . Assuming again independent uniformly distributed *p*-values under the global null, the test statistic is normally distributed, $T^{S}∼N(0,1)$ .

It has been shown^24^ that the closures of the Fisher combination test or the Stouffer test are in general not consonant. For example, if $p_{1}=p_{2}=0.06$ , for the Fisher combination test, $p_{\left\{1,2\right\}}=0.024$ but at significance level α=0.025 , no elementary hypothesis can be rejected with the local test. Also with Stouffer test, where $p_{\left\{1,2\right\}}=0.014$ , the test decisions are not consonant.

*Omnibus test*. To apply the Omnibus test^6^ for the global null hypothesis first the *p*-values $p_{1},\ldots ,p_{m}$ are sorted according to their size, $p_{\left(1\right)}\leq \ldots \leq p_{\left(m\right)}$ , and transformed with a monotonously decreasing function h . Then, cumulative sums of the transformed p-values are computed,

$$S_{i}=\sum_{j=1}^{i}h(p_{\left(j\right)}),\;i=1,\ldots ,m$$

The sums are again transformed using the distribution function $G_{i}$ of $S_{i}$ under the global null hypothesis. The final test statistic for the global null hypothesis is then given by

(1) $$T^{O}=\max_{1\leq i\leq m}G_{i}(S_{i})$$

and the final test decision for the global null hypothesis is performed with the corresponding *p*-values from the test statistics. The distribution functions $G_{i}$ can, for example, be computed with Monte Carlo simulations, sampling independent *p*-values from the uniform distribution on [0,1] .

**Figure 1.**
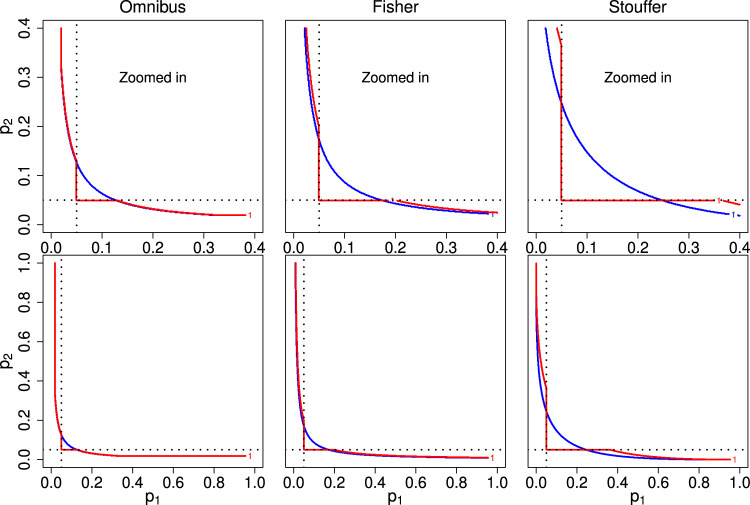
Rejection regions of the Omnibus, Fisher combination and Stouffer test of the intersection hypothesis $H_{\left\{1,2\right\}}$ and α=0.05 as a function of $p_{1}$ and $p_{2}$ (original test: blue line, modified test enforcing consonance: red line). The rejection region is below the corresponding line. The regions below and left to the black dotted lines show the rejection regions using an unadjusted α for the elementary p -values $p_{1}$ and $p_{2}$ . The modified consonant test is always bounded by the dotted lines in contrast to the blue line. The part below the blue line which lies above and right to the black dotted lines is the non-consonant area meaning the global test rejects, but none of the elementary. The first row shows the results for $p_{1},p_{2}\in \{0,0.4\}$ (zoomed in), the second for $p_{1},p_{2}\in \{0,1\}$

For the Omnibus test, different monotonously decreasing functions h were considered.^6^ h(p)=−logp turned out to be a good choice in terms of power compared to other considered transformations, which, however, leads to a non-consonant closed test. Consider, for example, a study where two hypotheses are tested with two stochastically independent test statistics and the elementary p -values $p_{1}=p_{2}=0.06$ are observed. For h=−logp , i=1,…,m , and α=0.05 , the global test with p -value $p_{\left\{1,2\right\}}=0.032$ can be rejected, but the closed test leads to no rejection in any of the two elementary tests as $p_{1}=p_{2}>\alpha$ . Also, for h(p)=z(p) and h(p)=1−p , the test is not consonant, whereas for the harmonic mean transformation h(p)=1/p for m=2 , $p_{\left\{1,2\right\}}=0.069$ and thus the global test is not rejected. We show in the Supplemental material (Figure 1) that the Omnibus test with h(p)=1/p is consonant for m=2 .

### 3.1. A procedure to construct consonant closed tests

We propose an algorithm to construct a consonant closed testing procedure based on combination tests. For this purpose, we modify the original combination tests for the intersection hypothesis tests such that a rejection is not possible if no rejection of an elementary null hypothesis occurs and then shift its rejection region to exhaust the local significance level α as much as possible. The resulting modified consonant test has an equal or increased power compared to the original non-consonant test.

Assume for each intersection hypothesis $H_{L}$ with index set L⊆M , a combination test with test statistic $T_{L}=f_{|L|}(p_{L})$ taking values in [0,1] is defined (where a low value of $T_{L}$ implies that the null hypothesis cannot be rejected whereas a higher value implies rejection of the null hypothesis). We assume the combination functions $f_{l}:[0,1{]}^{l}\to [0,1]$ , l=2,…,m satisfy

1.
$f_{l}$ is in each component nowhere increasing and
2.
$f_{l}$ is symmetric (e.g. for $L=\{1,2\},f_{2}(\{p_{1},p_{2},\})=f_{2}(\left\{p_{2},p_{1}\right\})$ .

Examples of combination tests that can be written in the above form are the Fisher combination, the Stouffer, and the Omnibus test. For weighted procedures, when using unequal weights, however, the symmetry assumption (Assumption 2) is not satisfied.

Based on the original test statistics $T_{L}$ , which may lead to a non-consonant test decision, we construct tests for the intersection hypotheses that lead to a consonant closed test and aim to exhaust the significance level α . In Algorithm 1, for each intersection hypothesis $H_{L}$ , L⊆M={1,…,m}, we construct a modified intersection hypothesis test by specifying a corresponding p -value $p_{L}^{c}$ , which can be written as function of the elementary p -values $p_{i}$ , i∈L . These p -values are constructed by induction on the cardinality of the index sets L .


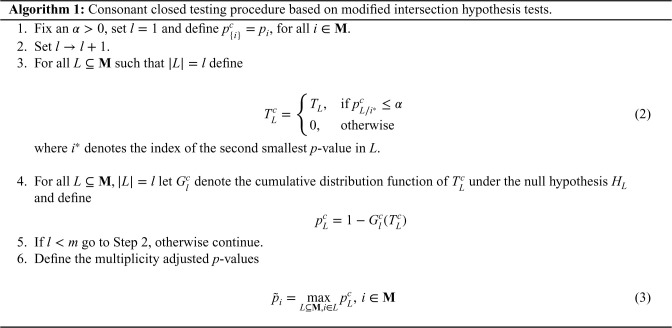


Some comments: (i) As shown below, the p -values $p_{L}^{c}$ can be written as a function of the elementary p -values such that $p_{L}^{c}=g_{|L|}^{c}(p_{L})$ and the functions $g_{|L|}^{c}$ depend on L only via the cardinality of the set L . (ii) As a consequence, also the cumulative distribution function $G_{|L|}^{c}$ of the test statistic $T_{L}^{c}$ , $G_{|L|}^{c}(T_{L}^{c})$ depends only on the cardinality of L , |L|=l .

In Theorem 1 (which is proven in the Appendix), we show that this algorithm gives a consonant closure test.

Theorem 1 Consider the test for the elementary null hypotheses $H_{i},i\in \text{M}$ , and let $p_{i}$ , i∈{1,…,m} , denote the corresponding elementary p -values, which have an independent joint distribution under $H_{\text{M}}$ . In addition, under all $H_{L}$ , L⊆M , the joint distribution of the $p_{i}$ , i∈L , is the same as under $H_{\text{M}}$ . Assume intersection hypothesis tests defined by combination functions satisfying conditions 1 and 2 have been defined and consider the corresponding closed test at some level α . Then the intersection hypothesis tests defined in Algorithm 1 define a consonant closed test that uniformly improves the original closed test for the elementary hypotheses, that is, the rejection region is nested.

Algorithm 1 can be applied to any non-consonant closed test based on combination tests that meet conditions 1 and 2. For example, for the Fisher combination, Stouffer or Omnibus test, we set $T_{L}=1-p_{L}$ . To generate the modified p -values, corresponding to the consonant test, the distribution functions $G_{|L|}^{c}$ of $T_{L}^{c}$ can be estimated by Monte Carlo simulation and the modified intersection hypothesis tests can then be performed with the one-sided p -values $p_{L}^{c}=1-G_{|L|}^{c}(T_{L}^{c})$ . The distribution function $G_{|L|}^{c}$ for independent test statistics can be estimated by Monte Carlo simulation with an iterative procedure. Starting with |L|=2 , a large number of random vectors of two independent p -values under the null distribution (uniformly distributed on [0,1] ) are generated and for each vector, the modified test statistic is calculated. The resulting set of test statistics then forms the null distribution of the test statistics for |L|=2 . For |L|=3 , random vectors of independent p -values under the null with three elements are generated and again the modified test statistics are calculated to create $G_{3}^{c}$ . Note that for this step also intersections of two hypotheses have to be considered and for this purpose, the procedure uses the distribution $G_{2}^{c},$ which has been generated in the previous step. This approach is applied iteratively for increasing |L| . To obtain reliable distributions, the number of random p -value vectors must be large. Below we illustrate the procedure for the test of m=2 and m=3 hypotheses for the Omnibus, Fisher combination and the Stouffer test.

#### 3.1.1. Examples

*Two hypotheses*. For m=2 , the modified test statistic (2) only needs to be computed for the global null hypothesis $H_{\left\{1,2\right\}}$ and is given by

(4) $$T_{\left\{1,2\right\}}^{c}=\min\left(T_{\left\{1,2\right\}},1_{\left\{p_{\left(1\right)}\leq \alpha \right\}}\right)$$

where (i) denote the indices of the ordered *p*-values and $1_{\left\{.\right\}}$ the indicator function. The corresponding one-sided modified p -value is given by $p_{\left\{1,2\right\}}^{c}=1-G_{2}^{c}(T_{\left\{1,2\right\}}^{c})$ .

If none of the elementary null hypotheses $H_{1}$ or $H_{2}$ can be rejected, the test statistic $T_{\left\{1,2\right\}}^{c}$ is zero. Consequently for both $p_{1}$ and $p_{2}$ larger than α , it always holds that $p_{\left\{1,2\right\}}^{c}=1$ and neither the intersection hypothesis $H_{\left\{1,2\right\}}$ nor an elementary hypothesis $H_{1}$ or $H_{2}$ can be rejected. If at least one elementary hypothesis can be rejected with the local test (i.e. $p_{1}\leq \alpha$ or $p_{2}\leq \alpha$ or both of them), the test statistic is not changed and $T_{\left\{1,2\right\}}^{c}=T_{\left\{1,2\right\}}$ . However, the resulting p -value $p_{\left\{1,2\right\}}^{c}$ may differ from $p_{\left\{1,2\right\}}$ as the null distribution of the test statistic $T_{\left\{1,2\right\}}^{c}$ may differ from the distribution of $T_{\left\{1,2\right\}}$ . The distribution of the modified test statistic $G_{2}^{c}$ for m=2 under the null hypothesis has an atom at zero, for example, for α=0.025 (0.05), the test statistics is 0 with a probability of 95.06% (90.25%).

For the case m=2 , Figure 1 shows the rejection region of the intersection hypothesis test with the original combination test for the global null hypothesis (blue line) and the modified intersection hypothesis test leading to a consonant closed test (red line) for the Omnibus test with $h=-\log\;p_{i}$ , the Fisher combination test and the Stouffer test for $p_{1},p_{2}\in \{0,0.4\}$ (zoomed in) or $p_{1},p_{2}\in \{0,1\}$ and α=0.05 (one-sided). The regions below and left to the black dotted lines correspond to outcomes, where one of the local tests of the elementary null hypothesis rejects at level α .

For the original Omnibus test, the rejection region where the intersection hypothesis but none of the elementary null hypotheses can be rejected is smallest and the rejection region of the modified procedure can hardly be visually distinguished as the improvement is small. For the Fisher combination and the Stouffer test, the part of the rejection region of the intersection hypothesis test where non-consonant test decisions occur is larger and consequently, the differences between the original and modified rejection regions are more pronounced. For example, for $p_{1}=p_{2}=0.05$ and α=0.05 , the original as well as the modified Fisher combination test according to (2) result in (rounded) global p -values of 0.017. If the elementary p -values are slightly increased to $p_{1}=p_{2}=0.051$ , for the original test, $p_{\left\{1,2\right\}}=0.018,$ whereas for the modified test $p_{\left\{1,2\right\}}^{c}=1$ . On the other hand, looking at the tails, for $p_{1}=0.05$ and $p_{2}=0.2$ , the original global test is not significant at level α with $p_{\left\{1,2\right\}}=0.056$ , whereas for the modified test, $p_{\left\{1,2\right\}}^{c}=0.049$ . For the Stouffer test, the rejection region where non-consonant test decisions occur is largest and the improvement by the modified test is most pronounced. For $p_{1}=p_{2}=0.05$ and α=0.05 , $p_{\left\{1,2\right\}}=0.010$ and $p_{\left\{1,2\right\}}^{c}=0.010$ . For $p_{1}=p_{2}=0.051$ , $p_{\left\{1,2\right\}}=0.01,$ whereas for the modified test $p_{\left\{1,2\right\}}^{c}=1$ . Looking at the tails, for $p_{1}=0.05$ and $p_{2}=0.35$ , $p_{\left\{1,2\right\}}^{c}=0.048$ and $p_{\left\{1,2\right\}}=0.076$ .

*Three hypotheses*. For a closed testing procedure with M={1,2,3} hypotheses, first, the modified test statistics for all pairwise intersection hypotheses $T_{\left\{1,2\right\}}^{c}$ , $T_{\left\{1,3\right\}}^{c}$ , and $T_{\left\{2,3\right\}}^{c}$ as defined in (2) have to be computed. Then, the modified test statistic for the global null hypothesis is given by

(5) $$T_{\left\{1,2,3\right\}}^{c}=\min(T_{\left\{1,2,3\right\}},\min(1_{\left\{p_{\left\{(1),(2)\right\}}^{c}\leq \alpha \right\}},1_{\left\{p_{\left\{(1),(3)\right\}}^{c}\leq \alpha \right\}}))$$

where (1),(2),(3) correspond to the indices of the ordered p -values. Note that unless at least two of the modified p -values for the tests of the pairwise intersection hypotheses $p_{\left\{1,2\right\}}^{c}$ , $p_{\left\{1,3\right\}}^{c}$ , and $p_{\left\{2,3\right\}}^{c}$ are less than α , the modified test statistic $T_{\left\{1,2,3\right\}}^{c}$ is set to 0 and thus the hypothesis cannot be rejected.

**Figure 2.**
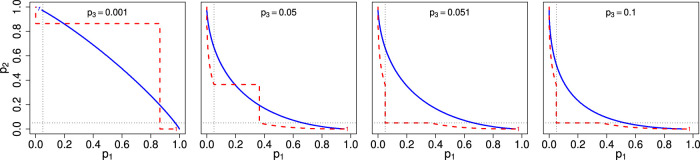
Rejection regions of the Stouffer test of the intersection hypothesis $H_{\left\{1,2,3\right\}}$ for m=3 , for varying $p_{1},p_{2}$ and several fixed values of $p_{3}$ , α=0.05 (red line for modified consonant test enforcing consonance, blue line for original test). The main difference between the red and blue lines is that if $p_{1}$ and $p_{2}$ are below the red line, this implies also a rejection of an elementary null hypothesis. However, for the original test (blue line), this implies only a rejection of $H_{\left\{1,2,3\right\}}$ . For the first two graphs, $p_{3}\leq \alpha$ , whereas for the third and fourth graphs with $p_{3}>\alpha$ an elementary hypothesis can only be rejected with either $p_{1}$ or $p_{2}$ below α . Therefore, for these cases, the red line is again bounded by the regions below and left to the black dotted lines, which indicate the unadjusted rejection regions of $H_{1}$ and $H_{2}$ (compare to Figure 1). For this visualization of the rejection area for m=3 , the red line can be also below the blue line as some rejection areas have to be shifted. This is because it also incorporates consonance testing of all intersections hypotheses of lower order as well.

For the example $p_{1}=p_{2}=p_{3}=0.05$ and α=0.05 , the original as well as the modified Fisher combination test according to (3) result in (rounded) p -values for the global null hypothesis of 0.006 (and 0.0022 for the respective Stouffer tests). If the elementary p -values are slightly increased to $p_{1}=p_{2}=p_{3}=0.051$ , for both original tests $p_{\left\{1,2,3\right\}}$ hardly changes, whereas for the modified tests $p_{\left\{1,2,3\right\}}^{c}=1$ . Figure 2 shows the region for the modified (red line) and the original closed Stouffer test (blue line) where the global test is significant at level α=0.05 for varying $p_{1}$ and $p_{2}$ at x - and y -axis and several fixed values of $p_{3}$ (see Figure 6 in the Supplemental material for results of the Fisher combination test). A small value of $p_{3}=0.001$ and large $p_{1}=p_{2}=0.8$ lead to rejection of $p_{3}$ with the modified test (with $p_{\left\{1,2,3\right\}}^{c}=0.041$ and $p_{\left\{1,3\right\}}^{c}=p_{\left\{2,3\right\}}^{c}=0.04$ ), whereas the original test is not rejected with $p_{\left\{1,2,3\right\}}=0.21$ (see Figure 2). When $p_{3}$ increases, simultaneously the rejection region of the modified test is shifted to the (0,0) coordinate. Compare, for example, the results for $p_{3}=0.05$ and $p_{3}=0.051$ : even though the change is only minimal, the “step” in the red line now vanishes. The reason is that for the latter ( $p_{3}=0.051$ ) the elementary p -value $p_{3}$ is not significant anymore and thus $p_{1}$ or $p_{2}$ have to be smaller than α in order to reject one of the elementary null hypotheses with the closed test.

#### 3.1.2. Shortcut

Given the exponential growth in the number of intersection hypothesis tests required in the closed testing procedure, for several multiple testing procedures efficient shortcuts have been developed.^14,25,26^ In particular, for combination tests, a shortcut in quadratic time has been proposed.^24^ Similarly, we can specify a shortcut quadratic in time for this procedure as well. We present the shortcut in two parts: First, in Algorithm 2, we construct consonant tests for intersection hypotheses $H_{L}$ , L⊆M , in an iterative manner requiring |L| steps. Then, in Algorithm 3, we define the shortcut for Algorithm 1, which involves tests of m intersection hypotheses. The tests of these intersection hypotheses are defined in Algorithm 2.


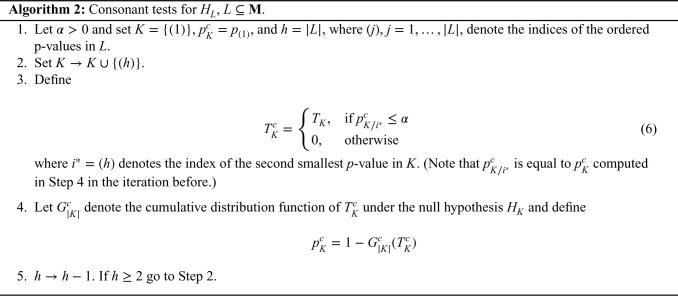



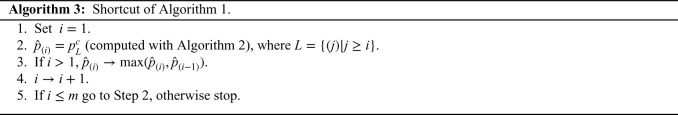


Note that the tests based on the multiplicity adjusted p -values ${\hat{p}}_{i}$ given in Algorithm 3 lead to the same test decision at level α as the adjusted p -values ${\tilde{p}}_{i}$ defined in equation (3) in Algorithm 1 (see Proposition 2 in the Appendix). The shortcut based on Algorithms 2 and 3 does not require testing all $2^{m}-1$ intersection hypotheses. Instead, Algorithm 3 only necessitates performing m intersection hypothesis tests. Furthermore, in constructing these tests, each iteration i involves creating m−i+1 additional hypothesis tests using Algorithm 2, resulting in a total of m(m+1)/2 intersection hypothesis tests. However, generally the p -values defined in Algorithm 3 are valid only at the level α used for constructing the consonant intersection hypothesis tests defined in Algorithm 2.

## 4. Simulation study

We performed a simulation study for m one-sample, one-sided z -tests of the means $\mu_{i}$ , i=1,…,m , of independently, normally distributed observations with known variance $\sigma^{2}=1$ . The hypotheses $H_{0i}:\mu_{i}\leq 0\;\text{ versus }\;H_{1i}:\mu_{i}>0\;i=1,\ldots ,m$ , are tested controlling the FWER in the strong sense at one-sided level α=0.05 . The number of hypotheses where the alternative holds is denoted by $m_{1}\leq m$ and all $m_{1}$ alternatives have the same mean effect size Δ . We assume equal sample sizes n=50 for each hypothesis and report the average power, defined as the proportion of rejected alternative hypotheses among all true alternatives, and the empirical FWER. All computations were performed using R.^27^

The simulation study has two parts:

1. Comparison of original and modified, consonant closed tests with the Omnibus test with log transformation, Fisher combination test, the Stouffer test, and the Hommel test^2^ (for details, see Table 1 and Supplemental material) as intersection hypothesis tests: the effect size Δ for the alternatives is chosen such that the power using a Bonferroni test with α/m is 1−β={0.2,0.3,…,0.9} for m={2,3,4,5} and one-sided α=0.05 .
2. Power comparisons of closure-based multiple testing methods: Bonferroni–Holm, Fisher combination test, Omnibus test (log and harmonic mean transformation of the p -values), Stouffer test, truncated test, harmonic mean p -value (HMP), Hommel test and Gou test are compared (see Table 1). The effect size Δ for the alternatives is chosen such that the power of the corresponding Bonferroni test is 1−β={0.5,0.7,0.9} with m={2,3,5,10} for one-sided α=0.05 .

**Table 1.** Closure-based multiple testing procedures used in the simulation study – assumptions of the original tests.

Test procedure	Combination	Independence	
(abbreviation)	function	assumption	Consonance^*^
Bonferroni–Holm^8^ (Holm)	Closure of Bonferroni adjustment: individual critical boundaries for each p -value	No	Yes
Hommel^2^	Closure of Simes test: individual critical boundaries for each p -value	Non-negative dependence	Partly (only for m=2 )^*^^21^
Gou^21^	Hybrid Hochberg–Hommel method	Yes	Yes
Fisher combination test^3^ (Fisher)	Sum of log p -values, see Methods	Yes	No^*^
Stouffer test^5^ (Stouffer)	Sum of z -scores	Yes	No^*^
Omnibus test with log transformation^6^ (Omnibus)	Cumulative sums of log-transformed p -values $-\log\;p_{i}$ , see Methods	Yes	No^*^
Omnibus test with harmonic mean transformation^6^ (Omnibus.h)	Cumulative sums of $1/p_{i}$ transformed p -values, see Methods	Yes	Yes
Harmonic mean p -value^20^ (HMP)	Harmonic mean of individual p -values, see Methods	No	Yes
Truncated product method^4^ (trunc)	Combination of p -values less than some cut-off	No	Partly^**^

^**^ For some testing procedures, consonance may only be given under certain restrictions. For more details, see Supplemental material. For testing procedures where the original version is non-consonant, modified procedures (indicated by ^*^) can be applied.

### 4.1. Comparison of consonant and non-consonant procedures

**Figure 3.**
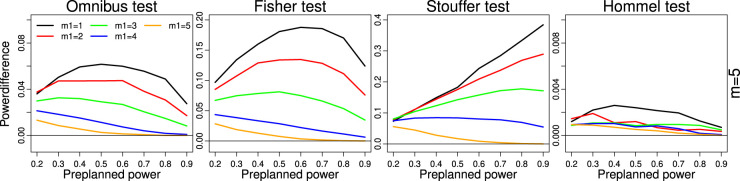
Gain in power in percentage points of the modified procedure at one-sided level α=0.05 for $m_{1}=\{1,\ldots ,m\}$ true alternative hypotheses, m=5 compared to the corresponding original procedure for the Omnibus, Fisher combination, Stouffer, and Hommel test. On the x -axis, the target power value using a Bonferroni adjustment α/m is shown.

Figure 3 shows the power improvement of the modified, consonant procedures compared to the respective original procedures for the Omnibus (log transformation of p -values), Fisher combination, Stouffer and Hommel tests, m=5 , $m_{1}=\{1,\ldots ,m\}$ , and α=0.05 (results for m<5 can be found in Figure 2 in the Supplemental material). Modified Hommel refers to the closed test where for each intersection hypothesis a Simes test is used and the consonance principle is applied. For each scenario, the effect size of the alternatives was chosen, such that a corresponding Bonferroni test would have a power of {0.2,0.3,…,0.9} , which is the value of the x -axis (the Bonferroni test was chosen to facilitate comparison between scenarios as the average power of the Bonferroni procedure does not depend on the value of $m_{1}$ , only on the value of m ). Different ranges on the y -axis are indicated for each method for better readability. The absolute power differences of the modified and the original procedures compared to the pre-specified power of the Bonferroni procedure for m={2,3,4,5} can be found in Figure 4 in the Supplemental material.

**Figure 4.**
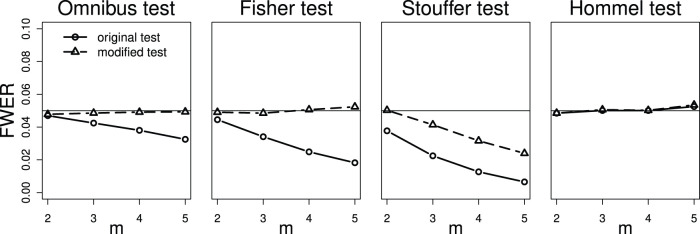
Actual familywise error rate (FWER) values of the modified and original procedures for the global null hypothesis with $m_{1}=0$ , m={2,3,4,5} and α=0.05 .

Generally, for the Omnibus, Fisher combination, and Stouffer tests for most scenarios, the modified test is strictly more powerful than the original test. Only for some scenarios with high pre-planned power and $m_{1}=m$ , the power is similar. The power differences increase with m and are larger for small $m_{1}=1$ . For almost all scenarios, the black curve, which depicts the scenario $m_{1}=1$ , shows the largest power difference. This is not surprising: for $m_{1}=m$ , and a sufficiently high effect size (depending on the pre-planned power) the null hypotheses will be correctly rejected in many simulation runs, even if some p -values are larger than α due to the combination of p -values. Hence, non-consonant scenarios will be rare also for the original test and thus the modified procedure cannot additionally increase the power substantially. If, however, $m_{1}=1$ , that is, only one true alternative hypothesis exists, with increasing m the influence of the one true alternative hypothesis on the test decision decreases whereas the influence of the true null hypothesis increases. Thus, the power of combination tests in the case of sparse alternatives ( $m_{1}/m$ is small) is smaller. Non-consonant scenarios may occur more often and the power of the combination test may improve when the consonant principle is applied to these scenarios. For the Stouffer and the Fisher combination test, this effect is larger because the test statistic for the global test sums over all p -values and thus the $m-m_{1}$ true null hypotheses have a large impact on the result (if some p -values are large others must be very small to obtain a significant result). Note, however, that for the Fisher combination test, this effect is less pronounced because of the logarithm in its formula, which gives higher weight to small p -values.

For $m_{1}=1$ and m=5 , for the Stouffer test, the power of the original test can be improved by approx. 40 percentage points and for the Fisher combination test by a maximum of 20 percentage points (Figure 3). According to Figure 1, for m=2, the Stouffer test has a larger non-consonant area (and smaller power values for many scenarios, as shown below) compared to Fisher combination or Omnibus tests and therefore more power can be gained with the modified test. For the Omnibus test and m=2 , the non-consonant area is small (see Figure 1) and accordingly the gain in power for the modified test statistic is also only small (see Figure 3, left column). With m , the improvement in average power increases and for m=5 and $m_{1}=1$ a maximum improvement of more than five percentage points can be observed.

For the modified Hommel test, the increase in power is only very small (note the small units on the y -axis). This is not surprising since the Hommel test is consonant for m=2 and ‘non-consonant rejections in Hommel’s procedure are rare if m is small’.^25^ Additionally, the Hommel test is already more powerful in scenarios with small $m_{1}$ compared to the Stouffer or the Fisher combination test. However, these are the scenarios, where in the simulations for the Stouffer or the Fisher combination test large power improvements were observed with the modified test.

Figure 4 shows the FWERs for the simulations from Figure 3 for $m_{1}=0$ and various values of m for the original (continuous lines) and the modified procedure (dashed line). For the Omnibus test, Hommel test and the Fisher combination test, the modified procedure approximately exhausts the alpha level for the global null hypothesis for the considered scenarios. For the Stouffer test, with increasing m>2 , the level is not fully exhausted. The reason lies in the condition that, for example, for m=3, two out of three combined modified p -values have to be significant. It has been shown^28^ that this condition is conservative for the z -test. Figure 5 in the Supplemental material shows the FWERs also for $m_{1}>0$ for the Omnibus, Fisher combination, and Stouffer test.

### 4.2. Power comparisons of closure-based multiple testing methods

Simulations were performed to compare the multiple testing procedures as shortly described in Table 1. More details on the methods can be found in the Supplemental material.

**Figure 5.**
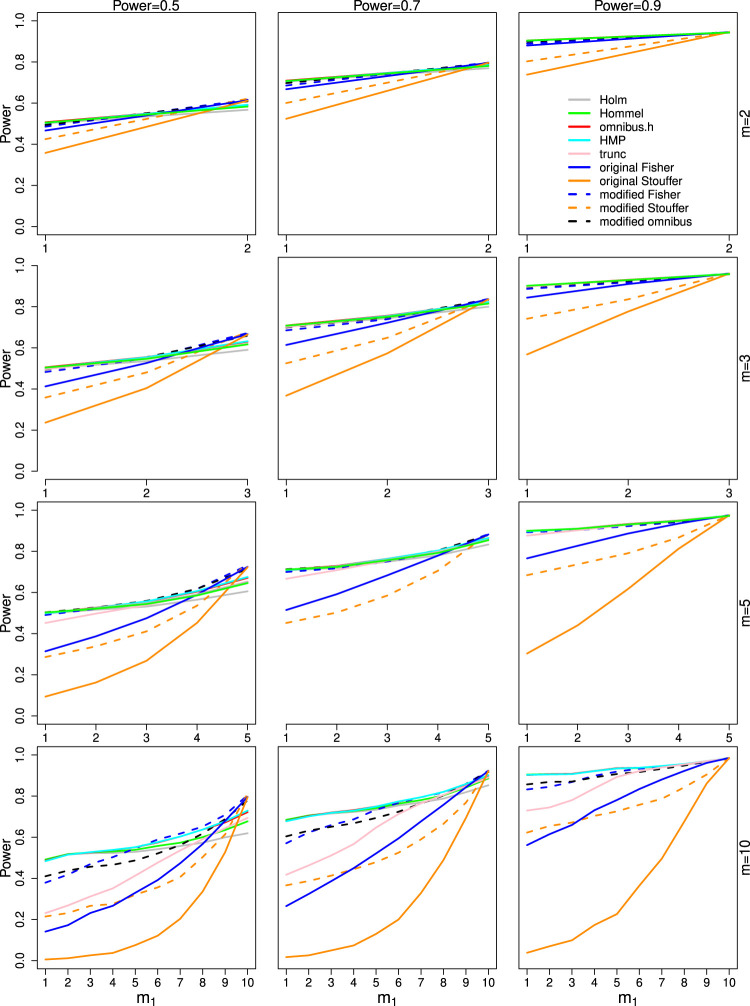
Average power as a function of the number of true alternatives $m_{1}$ ( x -axis) testing in total m={2,3,5,10} independent hypotheses (shown in rows) with one-sided α=0.05 . The columns are for targeted power of .5, .7, and .9 using a Bonferroni correction α/m .

Figure 5 shows the resulting average power values for m={2,3,5,10} and $m_{1}=\{1,\ldots ,m\}$ , α=0.05 . Effect sizes were chosen such that for the Bonferroni procedure a target power of .5, .7, or .9, was achieved for each m . For the Omnibus log test, the results of the modified procedure are shown, only for m=10 the results of the original test are given. For the Stouffer and Fisher combination test, both modified and original test results are shown. In the simulations, we considered the Hommel, modified Hommel and the Gou test and observed extremely small differences in power for these three methods. Thus, we only show the power values of Hommel’s test in Figure 5, whereas the results of the modified Hommel and Gou test are shown in Figure 7 in the Supplemental material.

It can be seen that no uniformly optimal test in terms of average power exists for all scenarios. The optimal test in the considered scenarios depends on the number of alternatives, the total number of hypotheses, and the effect size. For a large number of scenarios, many of the considered tests show similar power values. For $m_{1}=m$ for all considered scenarios, the modified Stouffer test has the largest power, followed by the Fisher combination test, whereas for decreasing $m_{1}$ , these two methods have partly much lower power values compared to the other methods. This is even more pronounced for larger m . Again the reason is that the test statistics for the intersection hypothesis tests of Stouffer and Fisher combination test sums over all p -values and thus the $m-m_{1}$ true null hypotheses have a large impact.

For smaller $m_{1}/m$ , in many scenarios, the Omnibus methods have the largest power values. For m=10 and small $m_{1}$ , also, for example, the Bonferroni–Holm, Hommel, modified Hommel, or Gou method yield high power values, whereas for $m_{1}=m$ , Bonferroni–Holm and Hommel methods show the lowest power values. This finding is in line with Henning and Westfall,^1^ who showed by simulations that the Hommel procedure is more powerful for larger m and smaller $\pi_{0}=1-m_{1}/m<0.9$ . Note that, for $m_{1}=1$ , also HMP shows rather comparable power values. In the Supplemental material, further investigations revealed that the modified Hommel has similar results as the Gou or the Hommel procedure.

As the number of true alternatives is unknown in practice, we tried to assess the overall behaviour of the tests for each considered value of m : For each method and each value of m , we calculated the difference to the target power of the Bonferroni procedure and averaged over the values of $m_{1}$ as well as over the different power values of target power .5, .7, and .9 (see Table 2). Positive values imply that the mean value is higher than the target power values, negative values vice versa. For all m≤5 , the Omnibus tests have the highest mean values, followed by the HMP test. For each m , the Stouffer method shows the lowest average power which is induced by the small power for small $m_{1}$ . We further looked at the maximum negative deviation from the target power values (Table 2). It can be seen that the modified Hommel, Gou, Bonferroni–Holm and Omnibus methods have the lowest maximum negative deviation, whereas the Stouffer test has the highest deviation.

**Table 2.** Averaged mean deviation and maximum negative deviation from the target power values .5, .7, .9 using a Bonferroni adjustment α/m for all values of m (averaged over $m_{1}$ and the target power values), α=0.05 . For Hommel, Stouffer, Fisher combination and Omnibus test, the modified procedures (indicated by ^*^) were applied. For the Omnibus test for m=10 , the results for the original test (without consonance) are reported due to running time in determining the null distribution.

m	Holm	Hommel^*^	Gou	Fisher^*^	Stouffer^*^	Omnibus^*^	Omnibus.h	HMP	trunc
**Averaged mean deviation**									
2	.030	.036	.036	.034	−.004	.038	.**039**	.038	.036
3	.039	.047	.047	.047	−.025	.**052**	.051	.050	.046
5	.035	.049	.046	.041	−.108	.047	.**050**	.049	.040
10	.046	.060	.058	.042	−.124	.030	.**064**	.**064**	−.035
**Maximum negative deviation**									
2	.001	.002	.002	−.018	−.103	−.007	.**003**	.001	.002
3	.000	.002	.002	−.018	−.173	−.003	.**003**	.002	−.002
5	−.005	−.002	−.002	−.010	−.251	−.**001**	−.004	−.008	−.051
10	−.018	−.**013**	−.**013**	−.119	−.327	−.091	−.023	−.021	−.278

HMP: harmonic mean p -value.  For each *m*, the values with the smallest deviations are highlighted in bold.

## 5. Application to real data sets

*Example 1*. Schlager et al.^29^ described the micro circulatory function of 896 children and adolescents for independent age groups (by tertiles) and performed subgroup analyses. For the variable baseline perfusion, a Mann–Whitney U -test was performed within each of the three age groups to compare males and females. We reanalysed the data with the Fisher combination, Stouffer, Hommel, and Omnibus test at level α=0.05 (see Table 3). Two vectors of one-sided p -values were generated (with equal effect size direction within each vector) and for each vector, the one-sided tests were performed at the half-significance level. All considered procedures (original and modified tests) led to the rejection of the hypothesis in the largest age group.

**Table 3.** The original two-sided p -values from the manuscripts and the corresponding generated one-sided p -values (in parentheses) for variables baseline perfusion and re-operation for each age group for the real data examples.

		Group 1	Group 2	Group 3
Example 1:	Two-sided p -value	0.97	0.09	<0.001
	One-sided p -values	(0.52, *0.49*)	(0.96, *0.045*)	(0.999, *<0.001*)
	Fisher	1 (0.49)	1 (0.11)	**<0.001 (<0.001)**
	Omnibus	1 (0.49)	1 (0.11)	<0.001 (<0.001)
	Stouffer	1 (0.49)	1 (0.11)	**0.0027 (0.0027)**
	Hommel	1 (0.49)	1 (0.09)	**<0.001 (<0.001)**
Example 2	two-sided p -value	**0.02**	**<0.01**	0.08*
	One-sided p -values	(*0.01*, 0.99)	(*0.005*, 0.995)	(*0.96*, 0.04)
	Fisher	0.037 (0.054)	**0.0247** (0.030^a^)	0.96 (0.96)
	Omnibus	**0.025** (0.026)	**0.014 (0.014)**	0.96 (0.96)
	Stouffer	1 (0.34)	1 (0.28)	1 (0.96)
	Hommel	**0.020 (0.020)**	**0.015 (0.015)**	0.96 (0.96)

Fisher combination, Omnibus, Hommel, and Stouffer tests were performed for each age group and adjusted p -values of the modified and the original test (in parentheses) are presented for the more promising direction (indicated by *italic font* for one-sided p -values), using one-sided α=0.025.

^a^ Non-consonant test result.

*Changed direction compared to other age groups.

*Example 2*. In a population-based cohort study, the long-term mortality after surgical aortic valve replacement with bioprosthetic (B) or mechanical aortic valve prostheses (M) in an Austrian population was investigated.^30^ We reanalysed the univariate results from Table 2 in Traxler et al.^30^ for the primary outcome death and the secondary outcomes re-operation, heart failure, myocardial infarction and stroke in the age groups <50 years, 50 to 65 years, and >65 years for the comparison of B versus M with Stouffer, Fisher combination, Hommel, and Omnibus test. As some of the p -values have not been reported such as the one-sided ones, all p -values needed (with three digits) have been provided by the authors^30^ on request. The original and modified combination tests were performed for both one-sided p -values at the half-significance level of 0.025. Table 3 shows the adjusted p -values and the results from the combination tests for the endpoint re-operation (see Supplemental material for other endpoints). In the second age group, the comparison B versus M is significant with the modified Fisher combination test, but not with the original test (here, the global test was significant, but the closed test did not reject). The Omnibus test and the Hommel test reject also the second age group with the modified and the original test and additionally reject the first age group (the Hommel test both with the original and the modified, the Omnibus test only with the modified test). The Stouffer test, however, rejects no age group for re-operation.

## 6. Discussion

We propose a general principle to construct consonant, closed multiple testing procedures. For the new, modified procedure, the original (non-consonant) test statistic is modified in order to shift the rejection region of the global test so that rejection of the global test is not possible if no rejection of a local test occurs such that the overall error level α is better exhausted. This principle is applied within the whole closed test. We also show how to construct multiplicity adjusted p -values based on the modified closed testing procedure. We prove that the modified closed testing procedure enforcing consonance improves the power uniformly compared to the corresponding original (non-consonant) closed testing procedure. We illustrate the method with various procedures and show by simulations that for some scenarios substantial power increases can be observed.

In the article, we only consider the modified procedure for combination tests based on independent test statistics. However, they can be extended to exchangeable p -values with known correlation, as, for example, in many-to-one comparisons. The algorithm and proof of Theorem 1 still apply, however, for the simulation of the null distributions, the p -values have to be sampled from the joint distributions taking the correlation into account. A direct generalization to general dependent test statistics is not possible due to the required symmetry property. Indeed the approach relies on exchangeability of the p -value distribution and on the fact that the distribution of the test statistics for the (intersection) hypotheses depends only on the number of hypotheses but not the set of hypotheses itself. In Figure 8 (in the Supplemental material), the Hommel, the modified Hommel and the Gou procedure are compared for positively dependent tests with known correlation for m=2 (equally correlated test statistics). Only minimal differences in power were detected for low and intermediate correlations. However, for larger correlations (e.g. ρ≥0.7 ) the modified Hommel procedure exhibited a larger but still modest improvement over the original Hommel test.

The proposed consonance principle has been developed for simple null hypotheses. Due to the monotonicity requirement on the combination functions, Type I error rate control of the procedure (Theorem 1) also holds for one-sided composite null hypotheses. In the case of some elementary null hypotheses, the effect size is strictly negative and positive for others, sum-based intersection hypothesis tests in general have a lower power than tests based on maximum statistics.

Consonance is a desired property when controlling the FWER in the strong sense.^13^ An alternative approach to adjust for multiple testing is the recently proposed simultaneous post-hoc false discovery proportion bounds.^23,25,31^ For such bounds, non-consonant rejections might improve the results obtained from the procedure. A comparison of the original and modified tests might be of interest regarding post-hoc false discovery proportion bound.

The approach of Romano et al.^11^ to improve non-consonant closed testing procedures in the case of two null hypotheses relies on the same principle as the approach considered in this article. They exclude sample points from the rejection region of the intersection hypothesis test that lead to non-consonance and adjust the critical value for the intersection hypothesis test to still exhaust the level α . Therefore, for m=2 , the procedure considered here, applied to the test statistics in Example 4.1 in Romano et al.^11^, results in the same consonant closed testing procedure. As shown in Romano et al.,^11^ this test is maximin optimal in the considered scenario. The procedure considered in this article extends to the case of m≥2 null hypotheses. The improved consonant testing procedure is obtained by induction in the number of elementary hypotheses included in the intersection hypotheses. In each step, to consonantize the intersection hypothesis tests, sample points leading to non-consonance are removed from the rejection region and the critical value of the intersection hypothesis test statistics are adjusted accordingly. However, for more than two hypotheses, this approach, in general, will not lead to optimal tests. This can be seen from the example of the Stouffer test. While the improved consonant procedure still uniformly improves the non-consonant test, it does not fully exhaust the level α (see Figure 4).

The methods described in this article can be used for several objectives. One aim might be replication studies, where, for example, a clinical trial or an experiment is replicated several times on independent observations.^32^ Another aim might be analysis of disjoint subgroups as shown in the real data set examples. In these applications, a high proportion of true alternatives and consequently several rejections might be expected.

We investigated the proposed approach in the context of clinical trials where, for example, treatment groups or subgroups are tested. In this setting, the number of hypotheses is typically small and thus simulations for m≤10 for the Stouffer and Fisher test and m≤6 for the Omnibus test (as the Omnibus test is computationally more demanding due to the simulation of the null distribution of the test statistic of the original test) were performed. Of course, comparisons for a larger number of tests can be performed with the proposed methods using the discussed shortcut.

In this article, a thorough simulation study was performed to compare several combination tests. P -value combination tests in a closure setting have been investigated, for example, in adaptive designs for clinical trials,^33,34^ where in each stage, p -values are calculated and a p -value combination test is then performed. Bauer and Kieser^33^ or Kieser et al.^34^ developed an adaptive procedure based on the Fisher combination test, whereas Lehmacher and Wassmer^35^ investigated the use of the Stouffer (or inverse normal) test to combine stages. In the considered simulation scenarios, the modified Stouffer and Fisher combination test were only superior for the case $m_{1}=m$ , but had rather small power values compared to other methods for small $m_{1}$ . This has already been noted by Henning and Westfall,^1^ who claimed that for independent p -values closure-based tests “perform terribly” unless the proportion of alternative hypotheses among the original set of hypotheses is extremely high. They further state that the Fisher combination test is only superior compared to Hommel for a smaller number of hypotheses < 20 and smaller $\pi_{0}=1-m_{1}/m$ , Stouffer test or truncated product method is only superior for $\pi_{0}=0$ . They thus recommend the Hommel test in comparison to the Fisher combination test or truncated product method in all scenarios, but for small $\pi_{0}=0.1$ and m<20 . However, in our simulations, we focused on settings with a maximum of m=10 hypotheses, commonly encountered in clinical trials. In this article, we show by simulations that the gain in power for the modified Fisher combination or Stouffer test compared to the original test for $m_{1}=1$ (and several values of m ) is surprisingly high. This is in contrast to the (modified) Hommel procedure where the increase in power is only very small, since there is no non-consonant rejection region for m=2 and the original test for small $m_{1}$ is already distinctly more powerful compared to, for example, the Stouffer test. The Omnibus tests, which are based on the cumulative sums of the transformed p -values, showed very good power properties for small and large values of $m_{1}$ . Note that these methods have not been considered in the work by Henning and Westfall.^1^ In the comparison with alternative approaches, the Omnibus methods are not always the best methods, but the differences to the respective best methods were rather small.

## Supplemental Material

sj-pdf-1-smm-10.1177_09622802241269624 - Supplemental material for A general consonance principle for closure tests based on p -values Supplemental material, sj-pdf-1-smm-10.1177_09622802241269624 for A general consonance principle for closure tests based on p -values by Sonja Zehetmayer, Franz Koenig and Martin Posch in Statistical Methods in Medical Research

## Appendix

To prove Theorem 1, we show the proposition below. Theorem 1 then immediately follows:

Proposition 1 Assume the assumptions of Theorem 1 hold. Then the p -values of the intersection hypothesis tests for all K⊆M can be written as function of the elementary p -values such that $p_{K}^{c}=g_{|K|}^{c}(p_{K})$ , where the functions $g_{|K|}^{c}$ are permutation invariant in the p -values $p_{i}$ , i∈K , nowhere decreasing in each component and depend only on the cardinality of K . Furthermore, for each K , the corresponding closed test for the family of hypotheses in K is consonant such that if $p_{K}^{c}\leq \alpha$ then

(7)
$$\max_{L\subseteq \text{K},i^{*}\in L}p_{L}^{c}\leq \alpha \;$$

where $i^{*}={\text{argmin}}_{i\in K}p_{i}$ (i.e. the closed test restricted to the hypotheses in K rejects the elementary hypothesis corresponding to the smallest p -value). Furthermore,

(8)
$$\max_{L\subseteq \text{K},i^{*}\in L}p_{L}\leq \alpha \Rightarrow \max_{L\subseteq \text{K},i^{*}\in L}p_{L}^{c}\leq \alpha \;$$

Proof. We proof by induction that Proposition 1 holds for all subsets K⊆M with |K|=k for k={1,…,m} . Base case: Let k=1 . Then the closed test for the families K={k},k={1,…,m} reduce to the elementary tests, and consonance and Assumptions 1 and 2 follow trivially. Induction step: Assume the proposition holds for the subsets with cardinality k−1 . We show that the proposition then also holds for the subsets K⊆M , with cardinality k . Let $T_{i}=f_{1}(p_{i})$ , where $f_{1}(p)=1-p$ . For each K⊆M with |K|=k let $T_{K}^{c}$ and $p_{K}^{c}$ be defined as in Steps 3 and 4 of the algorithm. By construction, the function $T_{K}^{c}:[0,1{]}^{k}\to [0,1]$ is a nowhere increasing function of the elementary p -values which is symmetric and is the same function for all K⊆M,|K|=k . Furthermore, by the independence assumption, the distribution of $T_{K}^{c}(p_{K})$ under $H_{K}$ is the same for all K⊆M,|K|=k and we denote the corresponding distribution function by $G_{k}^{c}$ . It follows, that $p_{K}^{c}$ can be written as a symmetric, nowhere decreasing function $g_{|K|}^{c}(p_{K})$ of the elementary *p*-values $p_{i},i\in K$ which is the same function for all K⊆M,|K|=k .
$p_{K\setminus \{i\}}^{c}\leq \alpha$ for all i>2 . As a consequence, for all intersection hypotheses $H_{L}$ , with L⊆M,|L|=k−1,1∈L , we have $p_{L}^{c}\leq \alpha$ . Now, since 1∈L∖{i},i=2,…,k , by the induction assumption also $p_{L^{*}}^{c}\leq \alpha$ for all $L^{*}\subseteq L\setminus \{i\},1\in L^{*},|L^{*}|<k-1$ . Now, (7) follows with $i^{*}=1$ . To prove (8), first note that the random variables $T_{K}$ dominate $T_{K}^{c}$ pointwise, which implies that for the distribution function $G_{|K|}$ of $T_{K}$ , we have $G_{|K|}\leq G_{|K|}^{c}$ . As above, w.o.l.g., let K={1,…,k} and consider an outcome where $p_{1}\leq p_{2}\leq \cdots \leq p_{k}$ . In addition, assume that $\max_{L\subseteq \text{K},1\in L}p_{L}\leq \alpha$ , that is, that the original closed test for the elementary hypotheses in K rejects $H_{1}$ . By the induction assumption, this implies that $p_{L}^{c}\leq \alpha$ for all L⊂K,|L|≤k−1,1∈L . By (2) this implies that $T_{K}=T_{K}^{c}$ . Because $G_{|K|}\leq G_{|K|}^{c}$ it follows that $p_{K}^{c}\leq p_{K}\leq \alpha$ . Together with the induction assumption, this shows (8). □

Proposition 2 At the level α used to define the consonant intersection hypothesis tests in Algorithm 1, the adjusted p -values ${\hat{p}}_{i}$ defined in Algorithm 3 lead to the same test decisions as the *p*-values ${\tilde{p}}_{i}$ , that is, ${\hat{p}}_{i}\leq \alpha \Leftrightarrow {\tilde{p}}_{i}\leq \alpha$ .

Proof. The proof is by induction in the hypothesis i . Assume without loss of generality (w.o.l.g.) that ${\tilde{p}}_{1}\leq {\tilde{p}}_{2}\cdots \leq {\tilde{p}}_{m}$ . Base case: (⇒) If ${\tilde{p}}_{1}\leq \alpha$ , then $p_{\text{M}}^{c}\leq \alpha$ and therefore by step 2 in Algorithm 3 ${\hat{p}}_{1}\leq \alpha$ . (⇐) If ${\hat{p}}_{1}\leq \alpha$ then $p_{M}^{c}\leq \alpha$ and by consonance and the permutation invariance and monotonicity of the p -value functions of the intersection hypotheses as defined in Proposition 1, it follows that ${\tilde{p}}_{1}\leq \alpha$ . Induction step: By the induction assumption ${\tilde{p}}_{i-1}\leq \alpha \Leftrightarrow {\hat{p}}_{i-1}\leq \alpha$ .
(⇒) If ${\tilde{p}}_{i}\leq \alpha$ then by the monotonicity of the p -value functions $g_{I}^{c}$ , we have ${\tilde{p}}_{j}\leq \alpha$ for all j<i . By the induction assumption, it follows that also ${\hat{p}}_{j}\leq \alpha$ for all j<i . In addition, since ${\tilde{p}}_{i}\leq \alpha$ implies that $p_{\left\{i,i+1,\ldots ,m\right\}}^{c}\leq \alpha$ it follows that ${\hat{p}}_{i}\leq \alpha$ .
(⇐) If ${\hat{p}}_{i}\leq \alpha$ then ${\hat{p}}_{j}\leq \alpha$ for all j≤i . By the induction assumption, for all intersection hypotheses $H_{L}$ with j∈L for some j={1,…,i−1}, we have $p_{L}^{c}\leq \alpha$ . In addition, since ${\hat{p}}_{i}\leq \alpha$ implies that $p_{I}^{c}\leq \alpha$ for I={i,…,m} . Now, by consonance and again by the permutation invariance and monotonicity of the p -value functions (as shown in Proposition 1) also $p_{L}^{c}\leq \alpha$ for all L∈M,i∈L and therefore ${\tilde{p}}_{i}\leq \alpha$ . □
