# Supplementary material for: A general consonance principle for closure tests based on p -values
Source: Stat Methods Med Res. 2024 Oct 23;33(9):1595–609. doi: 10.1177/09622802241269624 (PMC13254150; doi:10.1177/09622802241269624)
Supplement: sj-pdf-1-smm-10.1177_09622802241269624 - Supplemental material for A general consonance principle for closure tests based on p -values [file sj-pdf-1-smm-10.1177_09622802241269624.pdf]

Journal Title
XX(X):1–??

©The Author(s) 2016
Reprints and permission:
sagepub.co.uk/journalsPermissions.nav

DOI: 10.1177/ToBeAssigned
www.sagepub.com/
SAGE

Supplemental material: A general consonance principle for closure tests based on p-values

Sonja Zehetmayer, Franz Koenig, Martin Posch

This is supplemental material with supplemental results and additional plots for the manuscript "A general consonance principle for closure tests based on p-values". Authors: Sonja Zehetmayer, Franz Koenig, Martin Posch.

Center for Medical Data Science, Medical University of Vienna, Austria

Corresponding author:
Sonja Zehetmayer, Center for Medical Data Science, Medical University of Vienna, Austria
Email: sonja.zehetmayer@meduniwien.ac.at

Contents

Technical details on closure based multiple testing procedures **3**

Additional plots **8**

Hommel's procedures 9

Comparison of Hommel's procedures for positively dependent tests with known correlation **12**

Comparison with Bittman procedure **15**

Comparison with Romano procedure **18**

Additional results for real data example 2 **21**

Table 1. Closure based multiple testing procedures used in the simulation study - assumptions of the original tests.
** For some testing procedures consonance may only be given under certain restrictions. For more details, see supplemental material. For testing procedures where the original version are non-consonant, modified procedures (indicated by *) can be applied.

Test procedure (abbreviation)	Combination function	Independence assumption	Consonance*
Bonferroni-Holm [8] (Holm)	Closure of Bonferroni adjustment: individual critical boundaries for each p-value	no	yes
Hommel [9]	Closure of Simes test: individual critical boundaries for each p-value	non-negative dependence	partly (only for $m = 2$)* [5]
Gou [5]	hybrid Hochberg-Hommel method	yes	yes
Fisher combination test [3] (Fisher)	sum of log p-values, see Methods	yes	no*
Stouffer test [13] (Stouffer)	sum of z-scores	yes	no*
Omnibus test with log transformation [4] (omnibus)	cumulative sums of log transformed p-values $-\log p_i$, see Methods	yes	no*
Omnibus test with harmonic mean transformation [4] (omnibus.h)	cumulative sums of $1/p_i$ transformed p-values, see Methods	yes	yes
Harmonic mean p-value [16] (HMP)	Harmonic mean of individual p-values, see Methods	no	yes
Truncated product method [17] (trunc)	combination of p-values less than some cut-off	no	partly**

Technical details on closure based multiple testing procedures

In the following we give a more detailed description of the testing procedures used in the simulations, see Table 1. For Fisher, Stouffer and omnibus tests a more detailed description is included in the main manuscript.

Bonferroni-Holm procedure The most popular multiple testing procedure is the Bonferroni method, which rejects the global null hypothesis $H_1, \ldots, H_m$ if $\min p_i \leq \alpha/m$. Applying the closed testing principle to the Bonferroni method leads to the Bonferroni-Holm[8] correction: First the p-values are sorted according to their magnitude, $p_{(1)} \leq ... \leq p_{(m)}$ and individual critical boundaries are derived by $c_{(k)} = \alpha/(m+1-k)$ for each p-value $p_{(k)}$, $k = 1, \ldots, m$. Starting with the smallest p-value, each $p_{(k)}$ is rejected in case that $p_{(k)} \leq c_{(k)}$. If not, the procedure stops and all other p-values are not rejected. Both the Bonferroni and the Bonferroni-Holm methods are valid under any dependence structure of the p-values.

Truncated product method for combining p-values Zaykin et al. 2002[17] considered the product of all p-values that do not exceed some cut-off value τ, $0 \leq \tau \leq 1$ resulting in test statistic

$$T^W = \prod_{i=1}^{m} p_i^{I(p_i \leq \tau)}.$$

The critical values of this test can be found numerically. The truncated test is a consonant test if $1 - \sqrt{(1-\alpha)} \leq \tau \leq \alpha$, and thus consonance holds for $\tau = \alpha$[2].

Hommel's procedure Hommel's procedure[9] has been recommended by Henning and Westfall[7] as it is more powerful than several combination tests in particular for the case of small m_1/m. Hommel's procedure is based on the closed test of Simes for independent or non-negatively associated hypothesis tests[11,12]. Let j be the largest integer for which $p_{m-j+k} > k\alpha/j$ for all $k = 1,...,j$. If no such j exists, reject all hypotheses; otherwise, reject all H_i with $p_i \leq \alpha/j$ for $j = 1,\ldots,m$ and $i = 1,\ldots,m$.
As Hommel's method is only consonant for $m > 2$ (according to[5]), we also consider the modified procedure for the Hommel method.

Modified Hommel: Applying the closure of the Simes test in the proposed consonance principle generates modified Hommel.

Gou procedure Gou et al. (2014)[5] extended Hommel's procedure and derived consonant step-up procedures which the authors call "hybrid Hochberg-Hommel type step-up multiple test procedures". The proposed procedure controls the familywise error rate (FWER) under independence among the p-values, additionally under positive and negative dependence FWER control was shown by simulations. The method is implemented in the R-package *elitims*[6].

Table 2. Critical boundaries for the HMP test for $m = 2, 3, \ldots, 10$ (exact computations for $m \leq 3$ and simulated boundaries for $m > 3$).

m	critical bound
2	0.046029
3	0.044297
4	0.043260
5	0.042530
6	0.042016
7	0.041544
8	0.041224
9	0.040919
10	0.040668

Harmonic mean p-value (HMP) Wilson (2019)[16] introduced a test for the global null hypothesis based on the harmonic mean of the elementary p-values. The combined test statistic is defined by

$$T^{\text{HMP}} = \frac{m}{\frac{1}{p_1} + \cdots + \frac{1}{p_m}}. \tag{1}$$

The distribution of the test statistic and the computation of the critical boundary of T^{HMP} have been approximated with a Landau distribution. This method controls the FWER for independent and dependent hypotheses. As stated in[16] (and shown in Figure 1 for $m = 2$ for all combinations of p_1 and p_2 in the range $[0, 0.15]$), the HMP test is consonant. In the following, we derive an exact distribution of the test statistic for the HMP test for $m = 2$ and $m = 3$ and give some details on the usage of the HMP test as a closure-based approach.

HMP test: Exact distribution We derive an exact distribution of the test statistics for $m = 2$ and $m = 3$:

- The exact distribution of the test statistics for $m = 2$ is given by:

$$F(x, \alpha, m = 2) = \frac{2(x + \log(-1 + x))}{x^2} - \alpha \tag{2}$$

- The exact distribution of the test statistics for $m = 3$ is given by:

$$F(x,\alpha,m=3) = 1 - \frac{1}{3(-1+y)y^3}\{-6\log(-2+y)\left[3(-2+y)y + 2(-1+y)\log(-1+y)\right] \\ +(-1+y)\left[\pi^2 + 3(-3+y)y^2 - 6\log(-1+y)^2\right] \\ -24(-1+y)dilog\frac{1}{-1+y} + 12(-1+y)dilog\frac{-2+y}{-1+y}\} - \alpha$$

In Table 2 we give the boundaries for the HMP test with 6 decimal places. It follows that the null hypothesis can be rejected if $T^{\mathrm{HMP}}(p,m)$ is smaller than the corresponding boundary from Table 2. For $m < 4$, the boundaries are calculated exactly whereas for $m \geq 4$, the boundaries are derived via simulations.

To test the elementary hypotheses with the HMP test, different approaches are possible: (1) test for elementary hypotheses as proposed in Wilson et al.[16] with the R-package *harmonicmeanp*[15]; (2) test with exact boundaries for $m = 2$ and $m = 3$ and simulated boundaries for $m > 3$ for the test decision in the closure testing from Table 2. This approach better exploits the local level for small m and thus has higher power values. In the simulations described in the manuscript we applied approach (2) (exact boundaries for $m < 4$).

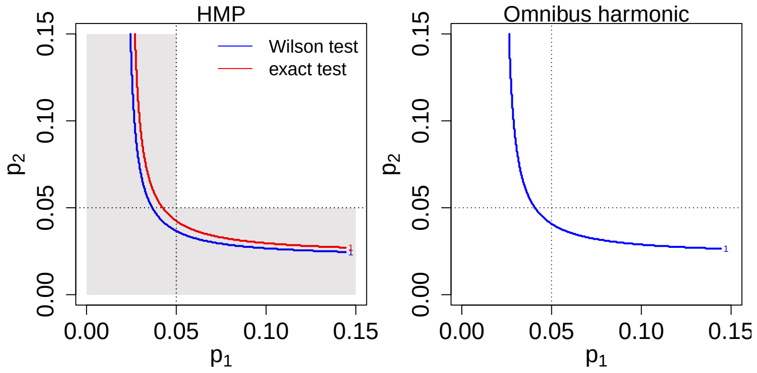

Figure 1. Left plot: The rejection region for the HMP test of the global (intersection) null hypothesis for $m = 2$ hypotheses is shown for the procedure based on the Landau distribution (blue lines) and on the exact boundaries (red lines) for $\alpha = 0.05$ (zoomed in for $p_1, p_2 \in [0, 0.15]$. The closed test only rejects if either p_1 or p_2 is below α and HMP is consonant for $m = 2$.
Right plot: Rejection region of intersection null hypothesis for omnibus test with harmonic mean of p-values for $m = 2$ (same scenario as for left plot).

Additional plots

The following graphs provide additional information on the calculations and simulations from the manuscript.

Fig. 3 in the manuscript shows the gain in power in percentage points of the modified procedure at one-sided level $\alpha = 0.05$ for $m = 5$, $m_1 = \{1, \ldots, m\}$ true alternative hypotheses compared to the corresponding original procedure for the Omnibus, Fisher, Stouffer, and Hommel test. Here the following Figs. 2 and 3 show the difference in power in percentage points of the modified (and the original) procedure compared to the target power of the Bonferroni adjustment α/m for $m = \{2, \ldots, 5\}$.

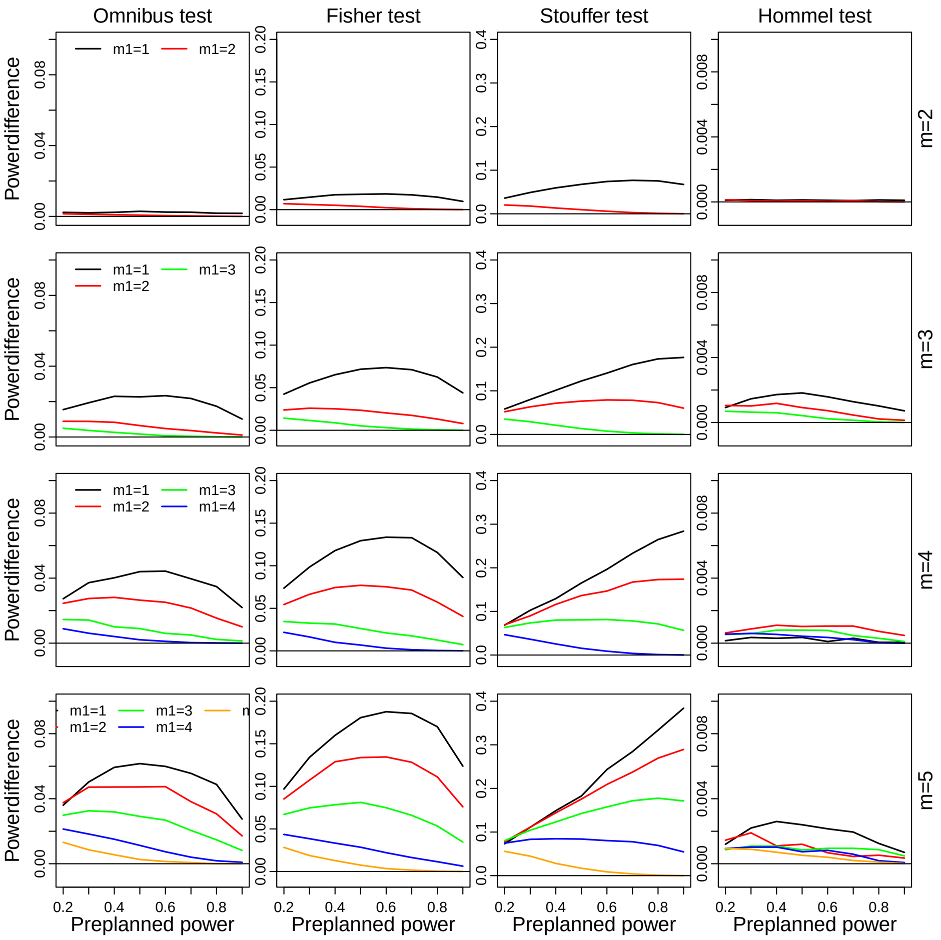

Figure 2. Gain in power in percentage points of the modified procedure at one-sided level $\alpha = 0.05$ for $m_1 = \{1, \ldots, m\}$ true alternative hypotheses compared to the corresponding original procedure for the Omnibus, Fisher combination Stouffer, and Hommel test (see columns). In the rows the results assuming testing $m = \{2, \ldots, 5\}$ hypotheses are shown. On the x-axis the target power value using a Bonferroni adjustment α/m is shown.

Hommel's procedures

Fig. 7 extends Fig. 5 from the main manuscript for the Hommel method, the Gou method (R library *elitims*[6]) and the modified Hommel.

For our considered scenarios we observed extremely small differences in power for these three methods. For some scenarios Gou and modified Hommel are slightly more powerful compared to Hommel, however the maximum improvement for the Gou is

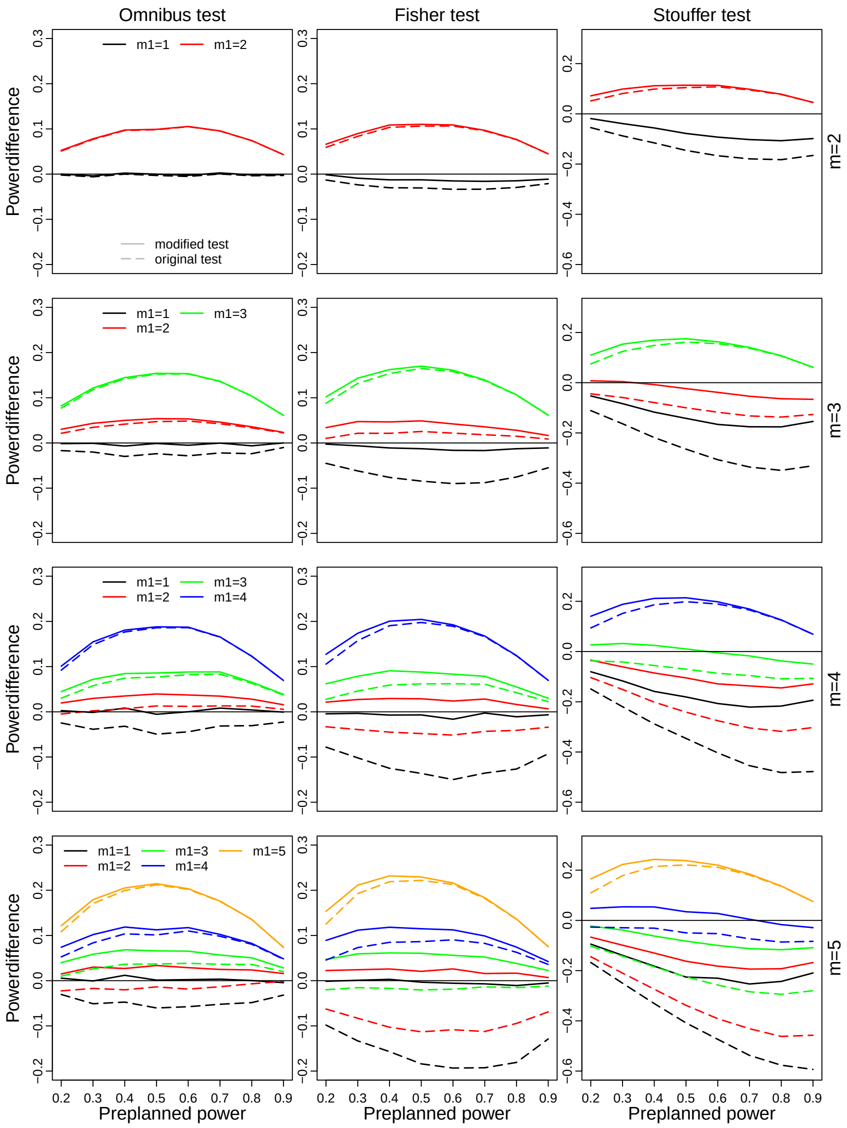

Figure 3. Difference in power in percentage points of the modified and original procedure at one-sided level $\alpha = 0.05$ for $m_1 = \{1, \dots, m\}$ true alternative hypotheses compared to the target power of the Bonferroni adjustment α/m for the Omnibus, Fisher and Stouffer test (see columns). Differences between modified and original test are shown in Figure 3 in the manuscript. In the rows the results are shown assuming testing $m = \{2, \dots, 5\}$ hypotheses. On the x-axis the target power value using a Bonferroni adjustment α/m is shown.

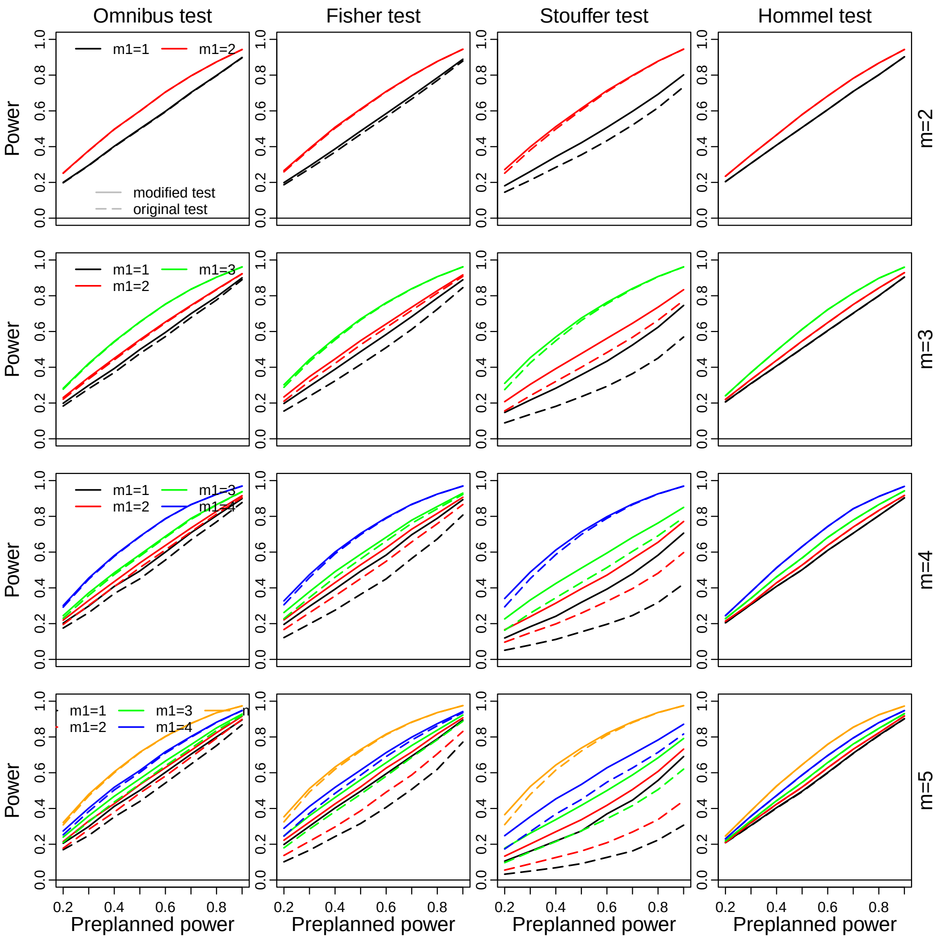

Figure 4. Actual average power for modified and original test procedures for scenarios from Fig. 2 from supplemental material and Fig. 3 from the manuscript.

0.0053 percentage points and 0.0028 for the modified Hommel. Note, however, that the modified Hommel (with consonance procedure) is very sensitive to its null distribution.

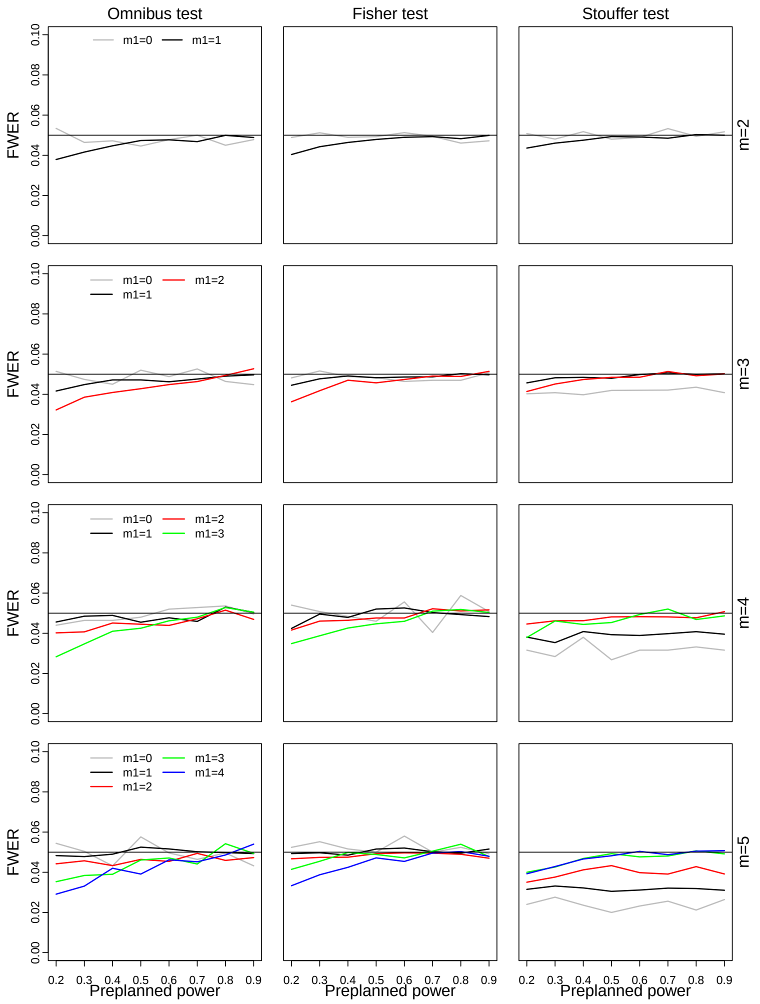

Figure 5. Actual FWER values for scenarios shown in Fig. 2 and 3 (and Fig. 3 in the manuscript) of the modified and original procedures for $m_1 = \{0, \ldots, m-1\}$ and $m = \{2, \ldots, 5\}$, $\alpha = 0.05$ (one-sided).

Comparison of Hommel's procedures for positively dependent tests with known correlation

For the case of $m = 2$ hypotheses, standard normally distributed data with a bivariate (equal) correlation of ρ, $\rho \in \{0, .8\}$ were generated for $n = 1$. The effect size of the first hypothesis was set to 2.5, for the second hypothesis several effect sizes were considered

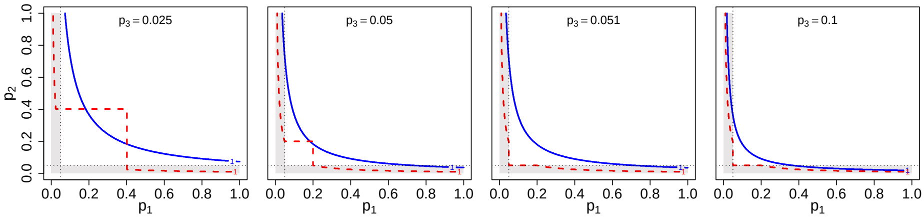

Figure 6. Rejection regions of the Fisher combination test of the intersection hypothesis $H_{\{1,2,3\}}$ for $m = 3$, for varying p_1, p_2 and several fixed values of p_3, one-sided $\alpha = 0.05$ (red line for modified consonant test, blue line for original test).

Table 3. Effect sizes of simulation study for Hommel, modified Hommel, and Gou procedure for positively dependent tests.

Scenario H_2	H_1	H_2
High effect	2.5	2.5
Moderate effect	2.5	2
Low effect	2.5	1

(see Table 3). Subsequently one-sided p-values were calculated for each hypothesis. To perform a multiple test with the modified Hommel procedure, for the simulation of the null distributions, the p-values were sampled from the joint distributions taking the correlation coefficient ρ into account.

In Fig. 8 results are shown for the Hommel, modified Hommel, and Gou procedure for $\alpha = 0.05$: marginal power for each hypothesis, the average power and the disjunctive power, i.e., the probability to reject at least one hypothesis. Note that for the marginal power no distinction between H_1 and H_2 is given in the presentation of the results. Again, only minimal differences in power were detected for low and intermediate correlations. However, for larger correlations (e.g., $\rho \geq 0.7$ the modified Hommel procedure exhibited a larger but still modest improvement over the original Hommel test.

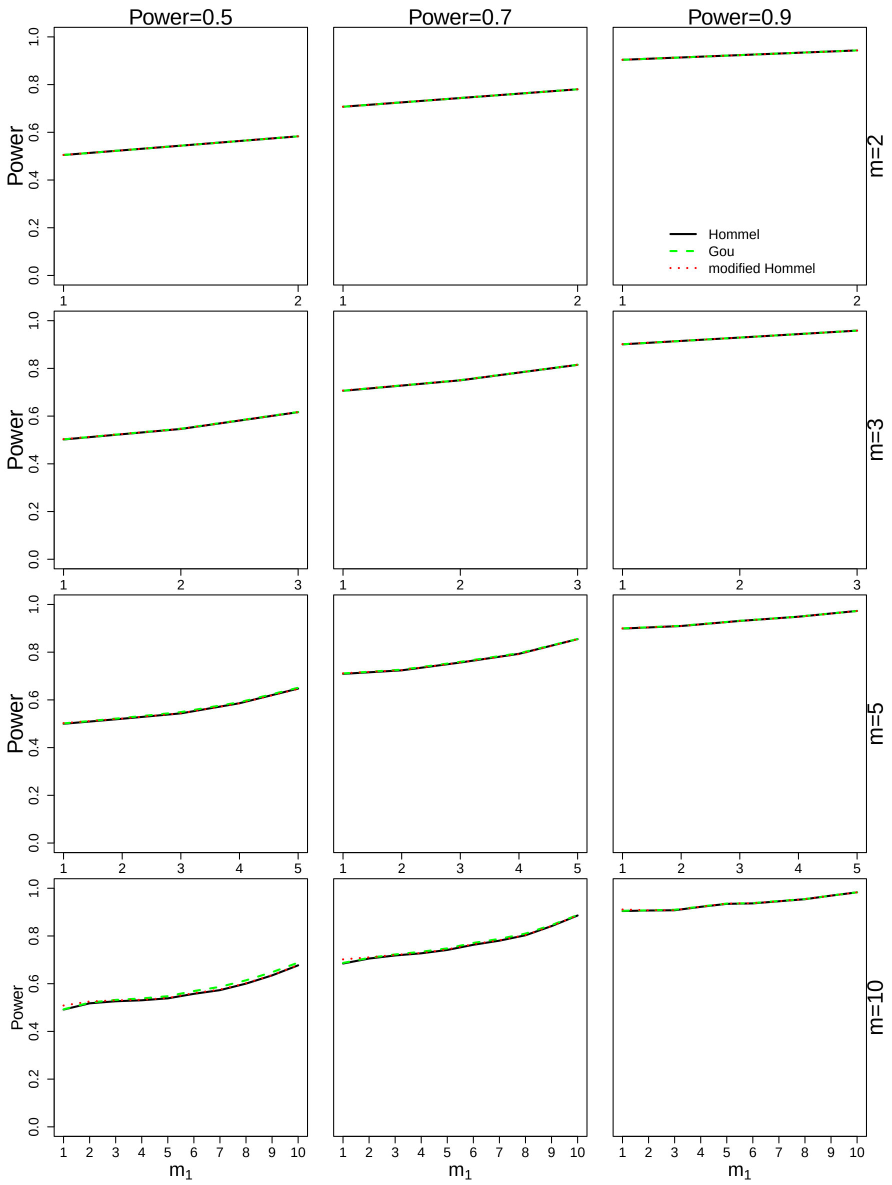

Figure 7. Average power as a function of number of true alternatives m_1 for Hommel, modified Hommel and Gou method (see Fig. 5 in the main manuscript). The columns are for targeted power of .5, .7, and .9 using a Bonferroni adjustment α/m

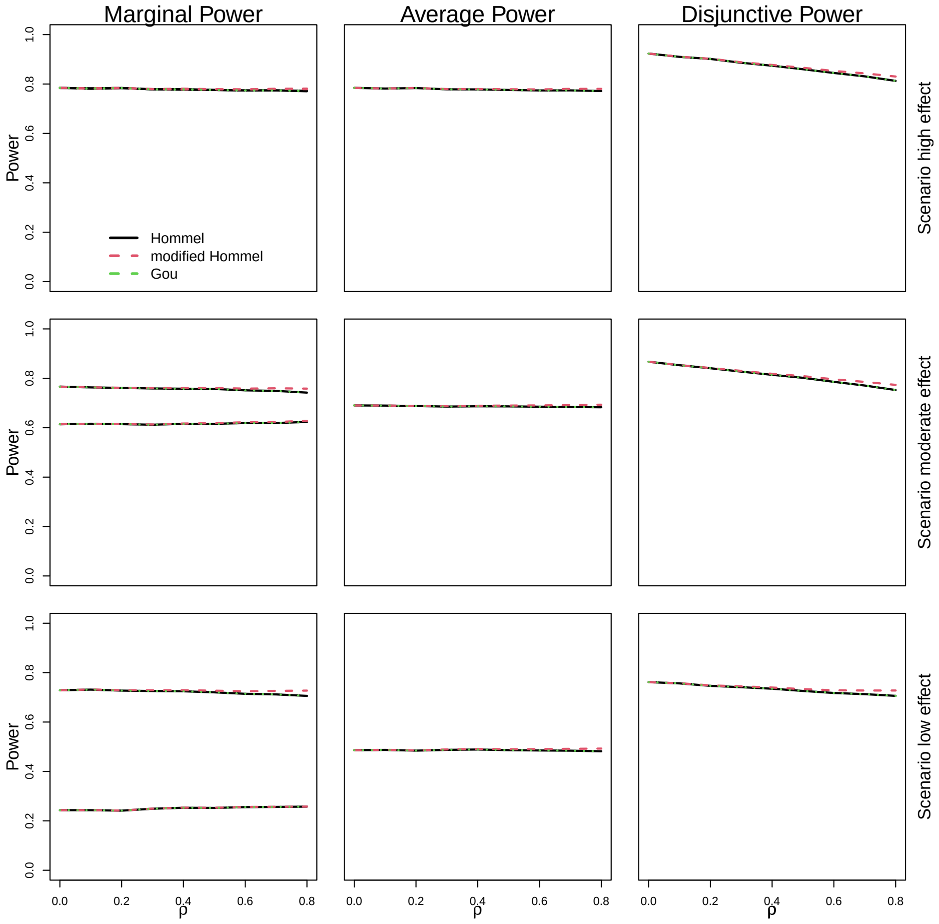

Figure 8. Marginal, average, and disjunctive power as a function of ρ for Hommel, modified Hommel, and Gou method for positively dependent tests.

Comparison with Bittman procedure

To compare the optimal consonant test of Bittman et al. (2009)[1] with the modified Stouffer test we reconstructed Fig. 1(a) from[1] on the z-scale. It can be seen that the rejection regions coincide in this case: The red line shows the rejection region for two one-sided normal means of the optimal consonant test of Bittman et al. (2009) with significance level 0.05 and $\rho = 0$. The test rejects for points left and below the red line. The black line shows the modified Stouffer test and it gives the same rejection region as

the optimal consonant test of Bittman et al. (2009). The green line shows the rejection region of the original Stouffer test.

The Bittman et al. (2009) procedure and the proposed algorithm have several differences: Our considered procedure is a general method which is based on p-values and applicable for several tests as shown in the manuscript (not only the Stouffer test). The method of Bittman et al. (2009) is only valid for normally distributed test statistics (exchangeable distribution for multivariate normal) whereas our method can be applied also for test statistics based on different distributions. Bittman's procedure is proposed for equal alternative hypotheses. Our procedure has no optimality principle.

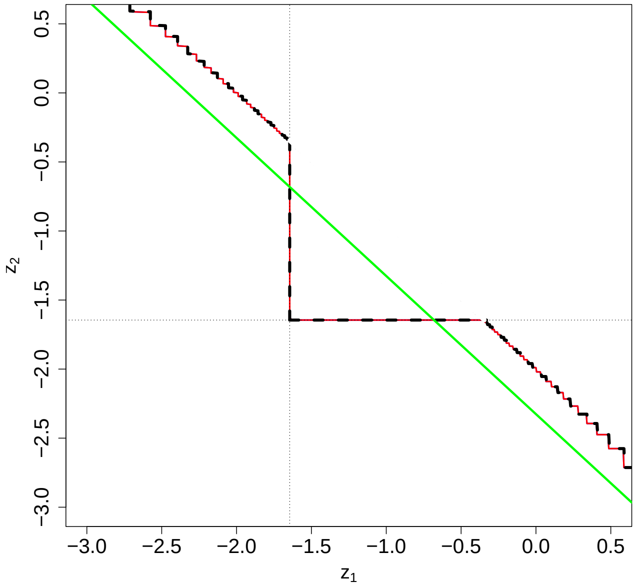

Figure 9. Reconstruction of Fig. 1 (a) of Bittman et al. (2009) [1] . The red line shows the rejection region for one-sided normal means of the optimal consonant test of Bittman et al. with significance level 0.05 and $\rho = 0$. The test rejects for points left and below the red line. The black line shows the modified Stouffer test and it gives the same rejection region as the optimal consonant test of Bittman et al. The green line shows the rejection region of the original Stouffer test

Comparison with Romano procedure

To compare the optimal test of Romano et al. (2011)[10] we reconstructed Figs. 3 and 4 of the original manuscript for two two-sided hypotheses, $\rho = 0$ and $\alpha = 0.05$ on the z-scale. The green square shows the unadjusted test, the red circle shows the optimal rejection region for $\varepsilon = .25$ of Romano et al. (2011). This rejection region is not consonant as also stated in Romano et al. (2011) (Note that the tests reject outside the circle/square). The blue square shows the rejection region for $\varepsilon = 3$, which now is a consonant multiple test. Note that these tests optimize the probability of rejecting at least one false elementary hypothesis. The modified Stouffer procedure is displayed by the black line. The comparison of the modified Stouffer and the Romano et al. (2011) procedure for $\varepsilon = 3$ shows that in scenarios where the absolute values of both z-values are high (≥ 1), the rejection region of the modified Stouffer is larger which means that more hypotheses are rejected. However, if one z-value lies close to 0, the rejection region of the Romano et al. (2011) procedure is larger.

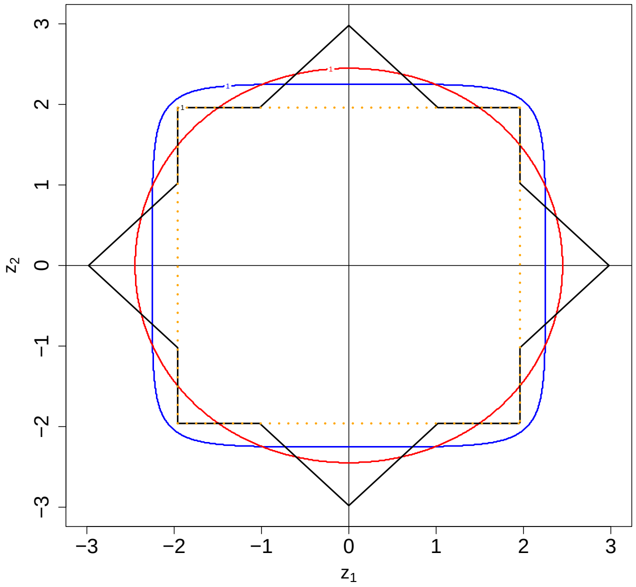

Figure 10. The yellow square shows the unadjusted test, the red circle shows the optimal rejection region for $\varepsilon = .25$ of Romano et al. whereas the blue square shows the rejection region for $\varepsilon = 3$. The modified Stouffer procedure is displayed by the black line. Note that the tests reject outside the circle/square.

The example in Romano et al. (2011)[10] is for two-sided test of two normal means. We thus adapted the original Stouffer procedure for the case of two-sided hypotheses. Instead of test statistic $T^S = (Z_1 + Z_2)/\sqrt{m}$ we set $T^S = (|Z_1| + |Z_2|)/\sqrt{m}$ where $Z_i = z_{1-p_i}$ and z_{1-p_i} denotes the $1 - p_i$ quantile of the standard normal distribution. A null distribution for this test statistic was generated using two-sided z-values.

Table 4. The original two-sided p-value from the manuscript and the corresponding generated one-sided p-values (in parentheses) for the real data examples 2[14]. Fisher combination, omnibus and Stouffer tests were performed for each age group and the results of the modified test and the original test (in parentheses) are presented; ”sig” means significant, ”ns” not significant.
[a] non-consonant test result
* changed direction compared to other age groups

		group 1	group 2	group 3
Death	two-sided p-values	0.06	**$<$0.001**	0.20
	one-sided p-values	0.97, 0.03	1, **$<$0.001**	0.9, 0.1
	Fisher	ns (ns)	sig (sig)	ns (ns)
	omnibus	ns (ns)	sig (sig)	ns (ns)
	Stouffer	ns (ns)	sig (sig)	ns (ns)
	Hommel	ns (ns)	sig (sig)	ns (ns)
Heart failure	two-sided p-values	0.73*	0.006	0.48*
	one-sided p-values	0.63, 0.37	0.003, 1	0.76, 0.24
	Fisher	ns (ns)	**sig (ns)**	ns (ns)
	omnibus	ns (ns)	sig (sig)	ns (ns)
	Stouffer	ns (ns)	ns (ns)	ns (ns)
	Hommel	ns (ns)	sig (sig)	ns (ns)
Myocardial infarction	two-sided p-values	0.17	0.02	0.32*
	one-sided p-values	0.92, 0.085	0.99, 0.010	0.16, 0.84
	Fisher	ns (ns)	ns (ns[a])	ns (ns)
	omnibus	ns (ns)	**sig (ns[a])**	ns (ns)
	Stouffer	ns (ns)	ns (ns)	ns (ns)
	Hommel	ns (ns)	ns (ns)	ns (ns)
Stroke	two-sided p-values	0.63	0.33	0.10*
	one-sided p-values	0.685, 0.315	0.835, 0.165	0.05, 0.95
	Fisher	ns (ns)	ns (ns)	ns (ns)
	omnibus	ns (ns)	ns (ns)	ns (ns)
	Stouffer	ns (ns)	ns (ns)	ns (ns)
	Hommel	ns (ns)	ns (ns)	ns (ns)

Additional results for real data example 2

We reanalysed the univariate results in a population-based cohort study where the long-term mortality after surgical aortic valve replacement with bioprosthetic (B) or mechanical aortic valve prostheses (M) in an Austrian population was compared (see Table 2 in[14]). Reanalysis was performed for the primary outcome death and the secondary outcomes reoperation (see manuscript), heart failure, myocardial infarction and stroke in the age groups $<$50 years, 50–65 years, and $>$65 years for the comparison of B versus M with Stouffer, Fisher combination and omnibus test. Results are shown in Table 4. More details on the methods can be found in the manuscript.

References

1. RM Bittman, JP Romano, C Vallarino, and M Wolf. Optimal testing of multiple hypotheses with common effect direction. *Biometrika*, 96(2):399–410, 2009.
2. E Dobriban. Fast closed testing for exchangeable local tests. *Biometrika*, 107(3):761–768, 2020.
3. RA Fisher. *Statistical Methods for Research Workers*. Oliver & Boyd, London, 1932.
4. A Futschik, T Taus, and S Zehetmayer. An omnibus test for the global null hypothesis. *Stat Methods Med Res*, 28(8):2292–2304, 2019.
5. J Gou, AC Tamhane, D Xi, and D Rom. A class of improved hybrid hochberg–hommel type step-up multiple test procedures. *Biometrika*, 101(4):899–911, 2014.
6. J Gou and F Zhang. *elitism: Equipment for Logarithmic and Linear Time Stepwise Multiple Hypothesis Testing*, 2019. R package version 1.0.4.
7. KSS Henning and PH Westfall. Closed testing in pharmaceutical research: Historical and recent developments. *Stat Biopharm Res.*, 7(2):126–147, 2015.
8. S Holm. A simple sequentially rejective multiple test procedure. *Scand J Stat*, 6:65–70, 1979.
9. G Hommel. A stagewise rejective multiple test procedure based on a modified bonferroni test. *Biometrika*, 75(2):383–386, 1988.
10. JP Romano, A Shaikh, and Wolf M. Consonance and the closure method in multiple testing. *Int J Biostat*, 7(1):1–25, 2011.
11. SK Sarkar. Some probability inequalities for ordered mtp2 random variables: a proof of the simes conjecture. *Annals of Statistics*, pages 494–504, 1998.
12. SK Sarkar and K Chang. The simes method for multiple hypothesis testing with positively dependent test statistics. *Journal of the American Statistical Association*, 92(440):1601–1608, 1997.
13. SA Stouffer, EA Suchman, and LC et al. DeVinney. *The American soldier: adjustment during army life. Vol 1.* Princeton, USA: Princeton University Press, 1949.
14. D Traxler, P Krotka, M Laggner, M Mildner, A Graf, B Reichardt, R Wendt, J Auer, B Moser, J Mascherbauer, et al. Mechanical aortic valve prostheses offer a survival benefit in 50–65 year olds: Autheartvisit study. *Eur J Clin Invest*, 52(5):e13736, 2022.
15. Daniel J. Wilson. *harmonicmeanp: Harmonic Mean p-Values and Model Averaging by Mean Maximum Likelihood*, 2019. R package version 3.0.
16. DJ Wilson. The harmonic mean p-value for combining dependent tests. *Proc Natl Acad Sci*, 116(4):1195–1200, 2019.
17. DV Zaykin, LA Zhivotovsky, PH Westfall, and Weir BS. Truncated product method for combining p-values. *Genet Epidemiol*, 22(2):170–85, 2002.
